# Can health promotion videos ‘go viral’? A non-randomised, controlled, before-and-after pilot study to measure the spread and impact of local language mobile videos in Burkina Faso

**DOI:** 10.1080/16549716.2019.1600858

**Published:** 2019-05-08

**Authors:** Tessa Swigart, Jennifer Hollowell, Pieter Remes, Matthew Lavoie, Joanna Murray, Mireille Belem, Rita Lamoukri, Souleymane Salouka, Kethakie Lamahewa, Roy Head

**Affiliations:** a Development Media International, London, UK; b Development Media International, Ouagadougou, Burkina Faso

**Keywords:** Mobile phones, videos, mass media, child health, rural

## Abstract

**Background**: Mobile phones present a new health communications opportunity but use of mobile videos warrants more exploration. Our study tested a new idea: to produce health promotion videos in languages for which films have never previously been produced to see if they were widely shared.

**Objective**: To investigate whether the novelty of films in local languages focusing on health messages would be shared ‘virally’ among the target population.

**Methods**: A non-randomised, controlled, before-and-after study was used to evaluate the reach and impact of the intervention. We gave short health promotion videos on memory cards to distributors in eight intervention villages. Ten control villages, where no video distribution took place were randomly selected. We conducted cluster-level difference-in-difference logistic regression to assess self-reported knowledge indicators. We calculated odds ratios for intervention relative to control at baseline and endline and p-values for the change in odds ratios.

**Results**: Seven hundred and eight mothers were interviewed across all villages at baseline and 728 different mothers and 726 men were interviewed in the same villages a year later in October 2015. At endline, 32% of women and 44% of men in the intervention arm had ever seen a film on a mobile phone in Lobiri, compared to 1% of women and 2% of men in the control arm. There was a significant increase in the odds of knowing about giving Orasel to a child with diarrhoea in the intervention area relative to the control area. Awareness of the need to take a child with fever or symptoms of pneumonia to a health centre increased in the intervention area, but not significantly.

**Conclusions**: Viral sharing of films on mobile phones has the potential to be an effective health promotion tool for communities whose languages are not served by existing mass media channels.

## Background

Mass media is often used in public health campaigns to promote healthy behaviours, increase knowledge, and influence social norms [1]. Mass media can be a relatively inexpensive way to reach a large target audience, and has been shown to change behaviours [2]. Public health interventions that use radio, TV, cell phones, and online media have had attributable success in health outcomes because such campaigns, if done well, based on extensive formative research, and broadcast with enough intensity, can reach a wide population while still targeting segmented audiences and stakeholders. This broad exposure can thus change the existing perceptions, attitudes, and norms of the given health problem, which in turn leads people to change their health-related behaviour [3–7]. Mobile phone technology has further extended the potential of mass communications, with an estimated 10 billion mobile phones in use at the end of 2016 [8]. In low- and middle-income countries (LMIC), mobile phone use has risen substantially in recent years [9] and many public health campaigns have already used mobile technology to promote a variety of health messages in low-resource settings [10]. A few of these campaigns have used videos in place of, or in addition to, other media such as radio and text messaging [11]. Videos provide a creative and potentially engaging way to capture attention while providing important health information. The possibility of mobile-to-mobile ‘viral’ sharing of videos, which has become more prevalent as mobiles make sharing fast and convenient, potentially provides greater exposure of campaign messaging at no additional cost to the campaign. A ‘viral video’ is defined as one that is rapidly shared with a multitude of people, nowadays most commonly through the Internet or other digitally transferable means [12]. A problem obstructing the use of this technology for public health purposes is a practical one: very few videos actually ‘go viral’, given that any given video must compete with millions of others on the Internet for attention [13]. This makes investment in such a dissemination strategy risky and less likely to yield an impact. Hence the potential of videos on mobile phones for public health promotion remains under-exploited.

An additional attraction of this new medium is that conventional media channels like radio and television do not reach all segments of the population. Some regions do not have radio stations or television signal, and some population groups live too far away from radio transmitters. Yet in some of these remote areas, mobile phone use is prevalent, making it a potentially useful channel to disseminate health promotion messages [14]. Research in this field has been limited: although there have been studies looking at the effects of mobile phone media on health, most are concentrated on mobile applications and text messaging, with few focused on videos [15,16].

From 2012 to 2015, Development Media International (DMI) conducted a mass media campaign to reduce child mortality in Burkina Faso, using radio spots developed and created in local languages [2]. The impact of the campaign was evaluated with a randomised controlled trial (RCT) [2]. During a trip to a rural radio station in Bogande, DMI’s research staff encountered Tindano Tibandiba Lasso, a mobile phone repair man, who had become well known for dubbing segments of famous movies into his local language, Gourmanche, and sharing these films on memory cards. They seemed to be spreading virally from phone to phone by people swapping memory cards and through Bluetooth.

The popularity of such films appeared to be due to the novelty of viewing them in the local language, in which (to our knowledge) no other films have ever been produced: the relatively few numbers of Gourmanche-speakers means that there has been no economic incentive for the local media industry to create content in that language. Indeed, films in Burkina Faso have primarily been produced in French (the lingua franca) and the two most dominant national languages (Mooré and Dioula). This pattern – reflecting the economics of the film and TV industries – is replicated around francophone Africa and across many LMICs.

A major opportunity therefore presents itself. In Africa alone, there are an estimated 1500–2000 languages [17], yet most of these languages have not been used in film or video production for economic reasons. If health promotion videos are the only videos ever made in a particular language, especially if they prove engaging and entertaining, they could potentially ‘go viral’. This strategy could reach new, mass audiences relatively cheaply and take advantage of the vast audio-visual potential of mobile phones.

We developed a pilot study to test this new communication strategy: producing short films on child health, in local languages, in areas with no or limited access to radio and television. Our study had two main objectives. One was to investigate whether short films in local languages, distributed via mobile phones, would be shared and spread virally among the target population, to see if this was a viable communication channel. The second was to see whether the films, if shared, had any impact on parental knowledge of appropriate treatment-seeking for malaria, diarrhoea, and pneumonia.

## Methods

### Study setting

Based on a survey of local media that DMI conducted in 2011 (M Lavoie, personal communication), we identified the Gaoua region of Burkina Faso, with a population of 320,000, as having among the lowest media penetration in the country (42% for radio, 5% for television). This setting allowed us to test the potential of viral video transmission in an area with very low traditional media consumption.

### Description of the intervention

DMI produced eight short videos of around three minutes in duration, in the Lobiri language spoken in the Gaoua region. The films were targeted at primary caregivers (mothers of children aged under five years) and promoted life-saving child health behaviours: giving oral rehydration salts (Orasel) to children with diarrhoea, seeking treatment for children with symptoms of fever (malaria), and seeking treatment for children with fast or difficult breathing (pneumonia). Behaviours were selected based on modelling using the Lives Saved Tool, to identify which would save the most under-five lives [18]. The films were distributed via memory cards in two waves to local distributors, across eight intervention villages. For the first wave in November 2014, we identified 80 local distributors from a range of backgrounds (shop-keepers, mobile phone repairmen, miners, farmers, etc.) and gave them memory cards that contained three films. The second wave took place in February 2015, when we gave five more films to the same local distributors. We provided a total of 356 memory cards to these distributors and encouraged them to share the films via Bluetooth or directly from the memory card.

### Design of intervention materials

Each film was designed to be an entertaining drama reflecting rural Burkinabe life, and scripts were created as film adaptations of some of the most successful radio short scripts identified from the previous study [2]. The scripts, and subsequently radio spots and then videos, were developed based on formative research with the target audience that explored the main barriers and facilitators to the target behaviour addressed in the messages. The scripts were designed to have an engaging storyline that captured the audience attention and were pretested before production to ensure they were motivating, relevant, entertaining, and believable to the audience.

### Study design

We used a non-randomised, controlled, before-and-after study design to test the reach and impact of the intervention. The evaluation included 8 intervention villages, where the videos were distributed, and 10 control villages, in areas with no distribution. We conducted a baseline and endline survey, in September 2014 and October 2015 respectively, measuring video exposure on mobile phones, as well as reported knowledge related to the key life-saving themes addressed in the videos. The questionnaire for this survey was based on an adaptation of a parental knowledge, attitudes, and practices questionnaire, focused on child health, that DMI, the London School of Hygiene and Tropical Medicine, and Centre Muraz research agency had developed and tested for the radio campaign RCT in Burkina Faso. This is described in detail elsewhere [19]. The survey for this pilot study was a shortened version with questions pertinent to the study objectives. The original survey had questions specific to radio listenership and exposure to the radio spots. For this study, these questions were replaced by piloted questions about exposure to mobile video viewing, and specifically on exposure to videos in Lobiri. Only health indicators from the original survey that were applicable to the outcomes for this study were maintained in the new questionnaire.

### Location and participant selection

We purposively chose 8 intervention villages using the following inclusion criteria: villages with fewer than 5000 inhabitants (to ensure a targeted rural population), villages without electricity, and villages at least 5 kilometres from a radio station. We then randomly selected 10 control villages within the same region that met the same criteria and were a minimum of 40 km from the nearest intervention villages.

The target population for the quantitative assessment was women of childbearing age (defined as 15–49 years), living in rural areas, who had at least one child under five years old. Most villages had fairly small populations so were divided into sections with the help of local guides. The enumerators first systematically listed all concessions in the village starting from a random point and enumerated households within each concession. A screening question was then administered to women of childbearing age within each household, and every fourth eligible woman was selected to participate. This was not a panel survey, so the endline survey included different participants than at baseline. Men were not originally included at baseline because women were considered to be the primary target audience for the messaging. However, at endline, we decided to include men of reproductive age because we thought we would be better able to measure exposure to mobile videos due to their higher rates of mobile phone ownership. To be eligible the men had to be in close contact to a child under five (father, grandfather, uncle, neighbour) but were not restricted to living in the same household as the child. The field work was conducted by Burkinabe researchers, each with a minimum of a bachelor’s degree and at least five years of research experience (MB, RL, SS), with supervision from PhD-level investigators (PR, JM, ML).

### Study sample size

Our two main outcomes were the number of people exposed to the viral videos at endline, and the change in knowledge for the three life-saving child health behaviours promoted in the films. No prior evidence exists on which to base assumptions for viral videos, so our sample size of 700 was based largely on financial and logistical constraints. A sample size of 700 was determined to be sufficient to estimate 20% exposure to the videos at endline with a confidence interval of *±*8% assuming a design effect of 2.

### Data analysis

We calculated frequency, means, and percentages for baseline and endline characteristics and for media use amongst the female cohorts, as well as for the male cohort at endline. We used a cluster-level difference-in-difference logistic regression analysis to assess the change from baseline to endline between control and intervention clusters in self-reported knowledge indicators. We report the odds ratios for intervention relative to control at baseline and endline and p-value for the change in odds ratio. All analyses were done with Stata (version 15) by researchers with a PhD in epidemiology (KL, TS, JH).

### Ethics

All data were kept in a locked and secure location. Ethical approval to conduct this research study was obtained from the Ethics Committee for Health Research in the Burkina Faso Ministry of Health. Informed written consent was obtained from all survey respondents, and any identifiable information was stored securely and shared with no one outside principal study personnel.

## Results

### Participant characteristics and access to radio, TV, and mobile phones at baseline compared to endline

For the baseline survey in September 2014, 708 mothers were interviewed across all control and intervention villages. An endline survey of 728 mothers and 727 men was then carried out a year later in October 2015. There were some differences in ethnicity and principal language spoken at baseline and endline for women and at endline for men between intervention and control areas, with the principal language in the intervention areas being Lobiri (96.1% at baseline and 97.1% at endline for women, 96% for men at endline). Lobiri was also the main language and Lobi the main ethnicity in control areas, but with a smaller majority (82.9% of women principally spoke Lobiri at baseline and 86.2% at endline; 86.1% at endline for men). At baseline, 21.9% of women had listened to the radio in the last seven days in the control groups compared with 17.9% in the intervention group. Access to radio and TV was low in both groups (63.7% and 69% did have a radio in the household in control and intervention respectively, and 96.8% and 93.3% did not have a TV). However, mobile phone use was high; in the control group, 80% of women had access to a mobile phone in their home or compound with 26.6% reporting ownership. In the intervention group, 83.6% had access to a mobile phone in their home or compound with 41.6% ownership reported (Table 1).

**Table 1.** Characteristics and media use of women at baseline and endline.

	Baseline				Endline			
	Control N = 377		Intervention N = 331		Control N = 385		Intervention N = 342	
	%	95% CI	%	95% CI	%	95% CI	%	95% CI
Age in years (mean)*	32.7	(31.7,33.7)	31.9	(30.6, 33.2)	32.8	(32.0,33.5)	31	(29.5,32.4)
**Length of time living in village**								
< 1 year	10.6	(5.8,18.4)	14.6	(9.5,21.6)	8.1	(6.4,10.0)	17.4	(13.2,22.3)
From 1 to 2 years	6.5	(4.1,10.0)	11.8	(9.5,14.6)	5.5	(3.3,8.8)	7.4	(4.9,13.2)
> 2 years	82.9	(76.9,87.7)	73.6	(68.6,78.1)	86	(82.3,89.0)	74.4	(65.4,81.7)
Don’t know	0	-	0	-	0.5	(0.07,3.7)	0.9	(0.3,2.7)
**Ethnicity**								
Dagara	7.9	(1.2,37.7)	0.3	(0.0,2.4)	4.2	(0.8,18.5)	0.3	(0.03,2.4))
Birifor	1.1	(0.5,2.3)	3.9	(0.9,14.9)	0.03	(0.0,1.0)	0	-
Dioula	-	-	-	-	0.3	(0.0,1.9)	1.5	(0.2,0.9)
Lobi	82.4	(61.8,93.1)	95.8	(85.5,98.6)	86.7	(72.2,94.3)	96.5	(92.2,98.4)
Mossi	6.8	(1.6,21.9)	0	-	6	(1.4,22.4)	0.3	(0.0,1.7)
Peulh	1.6	(0.9,2.8)	0	-	2.6	(0.9,7.3)	1.5	(0.5,4.3)
Other	0.3	(0.0,1.9)	0	-	0	-	0	-
**Language spoken daily**								
Dagara	7.9	(1.2,37.7)	0	-	4.2	(0.8,18.5)	0	-
Dioula	0.5	(0.1,3.8)	0.3	(0.0,2.4)	0.8	(0.01,5.4)	0.3	(0.0,1.7)
Fulfide	1.6	(0.9,2.8)	0	-	2.6	(0.9,7.3)	1.5	(0.5,3.45)
Lobiri	82.9	(62.2,93.5)	96.1	(81.1,99.3)	86.2	(70.8,94.2)	97.1	(92.2,98.9)
Birifor	0.5	(0.2,1.8)	3.6	(0.6,19.9)	0	-	1.2	(0.01,10.1)
Moore	6.5	(1.7,22.0)	0	-	6.25	(1.4,23.7)	0	-
**Access to radio**								
In compound	14.9	(9.0,23.8)	17.3	(10.7,26.8)	12.3	(0.7,20.8))	18.5	(14.5,23.4)
In household	21.4	(13.8,31.7)	13.7	(10.1,18.2)	36.8	(29.3,45.1)	29.1	(19.3,41.4)
No	63.7	(59.0,68.1)	69	(61.6,75.5)	50.9	(42.6,59.2)	52.4	(39.7,64.7)
**Last time listened to radio**								
Yesterday	10	(42.5,21.9)	7.6	(3.8,14.4)	14.9	(8.9,23.8)	11.5	(7.1,17.9)
In past 7 days	11.9	(8.9,15.8)	10.3	(4.5,22.0)	26.4	(17.0,38.5)	23.5	(12.0,40.8)
Prior to 7 days ago	62	(54.1,69.4)	57.1	(41.0,71.9)	33.9	(26.5,42.2)	41.2	(36.1,46.5)
Never	10.3	(4.9,20.4)	14.3	(7.2,26.2)	1.8	(0.6,6.6)	5.6	(1.8,15.8)
Unknown	5.7	(1.9,16.0)	10.6	(4.9,21.7)	23	(11.2,41.3)	18.2	(11.5,30.0)
**Access to mobile phone**								
In compound	28.2	(15.4,45.8)	34.7	(25.3,45.3)	67.1	(62.8,71.1)	63.5	(59.2,67.7)
In household	51.8	(36.3,66.9)	48.9	(34.7,63.3)	18	(14.7,21.9)	24.1	(18.4,31.0)
No	20.1	(14.7,26.7)	16.4	(8.9,28.2)	14.9	(10.7,20.3)	12.4	(9.3,16.3)
**Mobile phone ownership**								
Yes	26.6	(19.3,36.3)	41.6	(29.8,54.6)	32.6	(22.3,45.1)	44.4	(34.2,55.2)
**Access to television**								
In compound	1.4	(0.5,3.6)	3.7	(2.7,4.8)	4.1	(1.7,9.2)	4	(1.8,8.8)
In household	1.9	(1.0,3.5)	3	(1.5,6.1)	2.4	(0.7,8.0)	3.4	(1.5,7.3)
No	96.8	(94.0,98.3)	93.3	(90.2,95.5)	93.5	(84.3,97.4)	92.7	(86.7,96.0)
**Have you seen short films on a mobile phone?**								
Yes	43	(35.3,50.9)	45.5	(36.8,54.3)	53.7	(46.9,60.3)	65.4	(51.9,76.8)
No	56.8	(48.9,64.2)	54.2	(45.4,62.8)	46.4	(39.7,53.1)	34.6	(23.2,48.1)
I don’t know	0.3	(0.0,1.8)	0.3	(0.0,2.6)	0	-	0	-
**On whose phone did you view films? (multiple responses were possible)**								
My phone	22.6	(13.1,36.2)	25.3	(16.0,37.6)	29.6	(17.8,44.9)	24.7	(14.4,39.0)
My husband’s phone	24.5	(16.8,34.4)	12.7	(7.2,21.4)	16.5	(11.1,23.9)	11.7	(8.4,15.8)
Another person’s phone in compound	16.4	(9.7,26.2)	19.3	(13.6,26.7)	14.6	(8.9,22.9)	12.6	(7.0,21.5)
Another person’s phone in household	18.2	(11.5,27.6)	32.7	(24.9,41.4)	34.5	(23.1,48.0)	36.8	(29.8,44.4)
Friend’s	5.6	(2.3,13.4)	6.0	(3.1,11.2)	1.9	(0.6,6.0)	3.6	(1.4,8.8)
Neighbour’s	11.3	(5.3,22.7)	10.6	(6.3,17.5)	6.3	(3.5,11.1)	9.9	(3.7,23.5)
Other	47.8	(32.7,63.2)	61.3	(51.6,70.3)	56.8	(41.1,71.2)	63.2	(54.8,70.9)

*Missing for age at baseline N = 77 and N = 72 and endline N = 129 and N = 128 for controls and interventions respectively.

At endline, in the control group 41.3% reported listening to the radio in the past seven days compared with 35% in the intervention group. The other main media indicators remained largely unchanged at endline: a high proportion of women continued to report not owning a TV (93.5% and 93% for control and intervention groups respectively). The proportion of women owning their own mobile phone increased to 34% in the control group and 44.4% in the intervention group. At endline in the control group 54.5% of men reported owning a radio in their house or compound and 45.8% reported listening to the radio in the past seven days. In the intervention group, 50.3% reported owning a radio in their house or compound and 39.9% reported listening to the radio in the past seven days. TV ownership remained fairly similar between the two surveys of women, and also between men and women. Among men in the control group 66.1% owned a mobile phone compared to 63.6% in the intervention group (Table 2). Those that could view mobile videos on their phone was 28.3% among the control group and 36.5% in the intervention group (this indicator was not included in the survey of mothers).

**Table 2.** Characteristics and media use of men at endline.

	Control N = 380		Intervention = 347	
	%	95% CI	%	95% CI
**Age in years (mean)**	38.3	(34.3,42.3)	38.8	(34.7,42.9)
**Length of time living in village**				
< 1 year	2.6	(1.5,4.7)	2.6	(0.8,7.9)
From 1 to 2 years	2.4	(1.3,4.4)	6.1	(2.8,12.5)
> 2 years	89.7	(80.1,95.0)	85.3	(76.8,91.0)
Don’t know	5.3	(1.6,15.9)	6.1	(1.4,23.2)
**Ethnicity**				
Dagara	6.8	(1.1,32.1)	0.9	(0.1,6.4)
Birifor	0	-	3.5	(0.5,21.5)
Dioula	0.3	(0.0,1,2)	0	-
Lobi	85.5	(70.1,93.7)	95.1	(81.8,98.8)
Mossi	4	(1.3,11.3)	0.6	(0.2,2.1)
Peulh	3.4	(0.6,18.2)	0	-
**Language spoken daily**				
Dagara	6.6	(1.0,33.0)	0.3	(0.0,2.2)
Dioula	0.5	(0.1,2.0)	0	-
Fulfide	3.2	(0.4,19.3)	0	-
Lobiri	86.1	(70.1,94.2)	96	(80.4,99.3)
Birifor	0	-	3.5	(0.5,21.5)
Moore	3.7	(2.2, 6.1)	0.3	(0.0 2.0)
**Radio ownership**				
In compound	15	(7.7,27.1)	22	(11.7,37.4)
In household	39.5	(27.8,52.5)	28.3	(18.7,40.4)
No	45.5	(38.6,52.7)	49.7	(41.8,57.6)
**Last time listened to radio**				
Yesterday	24.5	(18.5,31,6)	24.3	(18.2,31.71)
In past 7 days	21.3	(16.8,26.6)	15.6	(12.5,19.3)
Prior to 7 days ago	33.9	(22.7,47.3)	39.6	(27.1,53.6)
Never	1.1	(0.2,4.6)	0.6	(0.1,2.4)
Unknown	19.2	(9.8,34.2)	19.9	(9.1,38.0)
**Access to mobile phone**				
In compound	20.8	(11.7,33.9)	34.1	(19.8,52.0)
In household	62.1	(46.7,75.4)	48.3	(32.6,64.2)
No	17.1	(11.1,25.3)	17.6	(13.2,23.2)
**Mobile phone ownership**				
Yes	66.1	(57.1,74.0)	63.6	(55.6,71.0)
**Able to watch videos/movies on their phone**				
Yes	28.3	(21.1,36.7)	36.5	(29.7,44.0)
**Access to television**				
In compound	1.3	(0.4,4.2)	2.6	(0.9,7.3)
In household	2.9	(1.0,8.2)	1.5	(0.6,3.5)
No	95.8	(91.2,98.0)	95.9	(90.4,98.3)
**Have you seen short films on a mobile phone?**				
Yes	21.6	(14.0,31.7)	45.9	(28.2,64.8)
No	78.4	(68.3,86.0)	54.1	(35.2,71.8)
**On whose phone did you view films? (multiple responses were possible)**				
My phone	67.1	(46.5,82.6)	45.6	(26.9,65.5)
My wife’s phone	0	-	1.3	(0.2,9.4)
Another person’s phone in compound	8.5	(4.0,17.0)	7	(4.6,10.4)
Another person’s phone in household	8.5	(2.2,28.0)	3.2	(1.3,7.5)
Friend’s	11	(7.2,16.4)	10.8	(6.7,16.9)
Neighbour’s	8.5	(3.1,21.9)	9.5	(3.7,22.4)
Other	18.3	(6.8,40.9)	10.1	(6.8,14.8)

### Mobile video viewing at baseline and endline

At endline, in intervention areas 60% of women had heard about films on mobile phones in their local Lobiri language compared to 9% of women in control areas. Among women in the intervention areas, 32% had ever seen a film on a mobile phone in Lobiri, compared to 1% in control areas. At endline a total of 44% of men in intervention areas and 2% in control areas had heard about Lobiri films on mobile phones. In the intervention villages 31% of men had ever seen a Lobiri language film on a phone, compared to 1% in control villages (Table 3).

**Table 3.** Lobiri film viewing at endline.

	Endline females				Endline males			
	Control N = 384		Intervention N = 341		Control N = 380		Intervention N = 347	
	%	95% CI	%	95% CI	%	95% CI	%	95% CI
**In the past 6 months, have you heard of films in Lobiri on mobile phones?**								
Yes	9.1	(3.9,19.8)	60.4	(42.7,75.8)	2.1	(0.8,5.3)	43.9	(24.0,66.0)
No	90.63	(80.4,95.9)	39.6	(24.2,57.3)	97.9	(94.7,99.2)	55.5	(33.9,75.3)
I don’t know	0.3	(0.0,2.3)	0	-	0	-	0.6	(0.1,2.4)
**In the past 6 months, have you seen any films in Lobiri on mobile phones?**								
Yes	0.5	(0.0,4.2)	32.3	(22.8,43.5)	0.5	(0.1,1.9)	31.1	(17.1,49.6)
No	99.5	(95.7,99.9)	67.7	(56.5,77.2)	99.5	(97.9,99.9)	68.9	(63.8,73.6)
**Do you have any of these short films on your phone now?**								
Yes	0	-	10	(4.8,19.8)	0	-	20.6	(13.9. 29.3)
No	100	-	90	(80.2,95.2)	100	-	79.4	(70.7,86.0)

### Knowledge at baseline and endline

Following the distribution of viral videos, there was a significant increase in the odds of knowing about giving Orasel to a child with diarrhoea in the intervention area relative to the control area. At baseline, participants in the intervention areas were less likely than participants in the control areas to know about giving Orasel to a child with diarrhoea (OR = 0.39) but by endline the situation had reversed: those in the intervention areas were more likely to know than those in the control areas (OR = 1.30). This change was significant (p < 0.001). Similarly, awareness among mothers of the need to visit a health facility if their child had a fever or if their child had symptoms of pneumonia was lower in the intervention group at baseline but again the situation had reversed by endline (baseline OR = 0.65, endline OR = 1.18 for fever, baseline OR = 0.64, endline OR = 1.04 for symptoms of pneumonia), but these changes between baseline and endline were not statistically significant. There was no improvement in mothers’ knowledge of issues that were not addressed in any of the Lobiri videos. For example, there was no difference in knowledge of the link between mosquitoes and the spread of malaria from baseline to endline (baseline OR = 0.65, endline OR = 0.64, p = 0.973) (Table 4).

**Table 4.** Cluster-level difference-in-difference logistic regression analysis showing baseline and endline odds ratio between control and intervention clusters for self-reported knowledge indicators.

	Baseline		Endline		
	OR	95% CI	OR	95% CI	P-value*
Do you know about Orasel tablets?	1.17	(0.64, 2.15)	1.72	(0.70, 4.24)	0.317
When your child has diarrhoea, what should you do?					
Give Orasel	0.39	(0.11,1.34)	1.3	(0.42,3.99)	0
When your child has a fever what should you do?					
Consult health centre	0.65	(0.25, 1.71)	1.18	(0.54, 2.59)	0.238
Consult community health agent	0.61	(0.19,1.99)	0.2	(0.03,1.17)	0.031
When your child has a cough and difficulty breathing what should you do?					
Consult health centre	0.63	(0.28,1.42)	1.04	(0.35,3.12)	0.337
Consult community health agent	0.62	(0.17,2.21)	0.28	(0.04,2.19)	0.221
What do you think causes malaria?					
Mosquitoes	0.65	(0.21,2.06)	0.64	(0.37, 1.12)	0.973
What do you think causes diarrhoea?					
Drinking non-drinking water	1.70	(0.63, 4.57)	0.5	(0.21, 1.22)	0.033
Dirty hands	0.67	(0.27,1.66)	1.83	(0.63,5.33)	0.146
Bad general hygiene	0.46	(0.20, 1.07)	0.5	(0.23,1.08)	0.885
Contact with someone sick	1.85	(0.29,12.01)	0.19	(0.01,2.78)	0.121

*P-value for change in odds ratio for intervention relative to the control from baseline to endline.

## Discussion

This pilot study was designed to test whether the innovative communication strategy of producing short health promotion films in local languages to be viewed and shared on mobile phones, in a location with low radio and TV penetration, would lead to the films being shared virally, potentially improving knowledge among the target audience. The results demonstrate that viral sharing of the campaign films did occur. Furthermore, some knowledge related to the treatment-seeking messages addressed in the films improved in the intervention group compared to the control group. Given the short time span of the study and the time it takes for films to circulate and be physically shared between phones, the reported increases in knowledge suggest that this communication approach could be a promising way of positively influencing knowledge, especially among marginalised populations that are difficult to reach with conventional media channels. Our findings provide evidence that the DMI films were virally shared; with around a third of all men and women reporting they had seen the Lobiri films in the intervention areas, and only 1–2% in the control areas. The proportion of those that had viewed any type of video on their mobile phone increased in both the control and intervention areas at endline (by 10.7% in the control areas and 19.9% in the intervention areas), which could reflect the increased number of those with access to video-capable mobiles. The fact that so few in the control areas reported viewing films in Lobiri gives further evidence supporting the viral sharing of our intervention videos. The evidence of peer-to-peer sharing is persuasive given the fairly low proportion of people who had access to a mobile phone on which they could view films (among men, only about a third). This figure is likely to increase in the future as more modern versions of mobile phones become predominant.

This study supports the conclusions of a study looking at viral sharing of media on mobile phones in urban India, which found that the desire for entertainment was enough motivation to surpass technological and socioeconomic obstacles in sharing videos virally [15]. Our videos were designed to be engaging and entertaining while also communicating important health messaging. According to the literature, the motivation to share digital information is only partly driven by how interesting and important the information is; it is also dependent on the sharer’s desire to seem helpful and/or knowledgeable, and how emotionally connected they feel to the content of the media [13,20]. Our results show, then, that if designed well, a mobile video intervention represents a new communication channel for reaching more marginalised populations who do not currently have access to radio or TV, as well as reaching those who do. To our knowledge, no other studies have investigated the potential of public health campaigns delivered via mobile phones using short entertaining films in local languages. Most have focused on other types of mobile phone interventions, namely text messaging; a 2013 review of mobile health approaches reported that the majority of mobile campaigns have used one-way text messages to transmit information, nudges, and appointment reminders [21]. Other studies note that the effect of mobile campaigns is hard to determine, due to a lack of outcome evaluations, especially in low-resource settings [10,21,22]. There is scope, therefore, for further research to test the effectiveness of mobile phone videos in changing behaviours and health outcomes. But the main impact of our own research may be practical: to identify a gap in the market for local language videos that the public health community can utilise. Future research may also be driven by practical developments: in our pilot study, the chosen distribution channels (SD cards and Bluetooth) seemed very effective, but as technology evolves it is worth exploring how mobile networks or community health workers, for example, could be used to push out content.

Will this opportunity last? While the economics of the TV and film industries are unlikely to change for the next decade or two, it is worth noting that the increased penetration of smartphones will bring access to video material from around the world, and also home-made content which may be in local languages. It will be interesting to see whether professional films produced in local languages maintain the same novelty in areas where there is more competition. Additionally, with the rising quantity of easily available media comes the risk of spreading incorrect and damaging information. As well as being entertaining enough to trigger significant peer-to-peer sharing, any health promotion video must be designed responsibly so that no false, misleading, or dangerous health information is communicated inadvertently.

There were some limitations to our study. The survey was a pre/post design of reported exposure and health knowledge. It is therefore difficult to determine with certainty that any changes in knowledge (or subsequently behaviours) were attributable to the video campaign alone. However, since we did use a control group, we can say that it is unlikely there were external factors influencing the intervention group that did not also influence the control group, so the differences between them from baseline to endline provide reliable evidence. Further research is needed over a longer period of time to examine the impact on health behaviour outcomes. We also did not measure the extent of the ‘virality’ of the videos. Our primary outcomes looked at the number of people who had seen our videos at endline, and those who had our videos on their mobile. Additionally, we did not assess the extent to which the videos spread outside the intervention villages where the distribution points were located. Further studies could explore exposure to the intervention messages in more detail, in particular how and why the videos were shared and the extent to which individuals viewed them multiple times. An endline survey of men was added after it was noted that mobile phone ownership was much higher among men than women. In future investigation we would recommend enlisting both men and women from baseline to endline. There were also differences in ethnicity and language spoken, with the intervention groups having a larger ethnic and language majority of Lobiri than the control groups. This was probably because the intervention villages were purposively chosen and then the control villages chosen randomly based on eligibility criteria. However, the control areas did contain a Lobiri majority, so we do not feel this would have affected the results significantly. Lastly, this was a relatively small pilot test, and a large nationally scaled-up study would add greatly to the evidence for the feasibility and effectiveness of this type of communication strategy.

## Conclusions

Our original hypothesis was that by exploiting the strong appetite for video content in local languages in rural Burkina Faso, particularly among audiences with low radio and TV penetration, we could create entertaining health content that would be shared from peer to peer on mobile phones and motivate improvements in knowledge. This pilot study has provided convincing evidence in support of this hypothesis and showed that local language videos can indeed ‘go viral’. Given the huge number of languages in LMICs which are under-served by the film and television industries, the potential of this approach as a health promotion tool appears to be considerable. Further research is needed to investigate the potential for this strategy to change behaviours and to be delivered at scale.

## Paper context

Mass media has been shown to be effective in changing health behaviours. Yet traditional mass media channels do not reach some rural or remote areas. Mobile phones present a new opportunity. This study suggests that producing health video content in local languages resulted in viral sharing of films on mobile phones and has the potential to be an effective health promotion tool for communities whose languages are not served by the existing mass media channels.
